# The Graft of Autologous Adipose-Derived Stem Cells in the Corneal Stromal after Mechanic Damage

**DOI:** 10.1371/journal.pone.0076103

**Published:** 2013-10-01

**Authors:** Xiao-Yun Ma, Hui-Jing Bao, Lei Cui, Jun Zou

**Affiliations:** 1 Department of Ophthalmology, Guanghua Integrative Medicine Hospital, Shanghai, China; 2 Department of Ophthalmology, Shanghai Sixth People’s Hospital, Shanghai Jiao Tong University, Shanghai, China; 3 Medical Technology and Engineering Institute, Henan Science and Technology University, Luoyang, Henan, China; Instituto Butantan, Brazil

## Abstract

This study was designed to explore the feasibility of using autologous rabbit adipose derived stem cells (rASCs) as seed cells and polylactic-co-glycolic acid (PLGA) as a scaffold for repairing corneal stromal defects. rASCs isolated from rabbit nape adipose tissue were expanded and seeded on a PLGA scaffold to fabricate cell-scaffold constructs. After 1 week of cultivation in vitro, the cell-scaffold complexes were transplanted into corneal stromal defects in rabbits. In vivo, the autologous rASCs-PLGA constructed corneal stroma gradually became transparent without corneal neovascularization after 12 weeks. Hematoxylin and eosin staining and transmission electron microscopy examination revealed that their histological structure and collagen fibril distribution at 24 weeks after implantation were similar to native counterparts. As to the defect treated with PLGA alone, the stromal defects remained. And scar tissue was observed in the untreated-group. The implanted autologous ASCs survived up to 24 weeks post-transplantation and differentiated into functional keratocytes, as assessed by the expression of aldehyde-3-dehydrogenase1A1 (ALDH1A1) and cornea-specific proteoglycan keratocan. Our results revealed that autologous rASCs could be one of the cell sources for corneal stromal restoration in diseased corneas or for tissue engineering of a corneal equivalent.

## Introduction

Corneal damage resulting from trauma, infection, dystrophy and corneal failure can lead to corneal opacification, visual impairment, and even blindness [1]. Up to now, it has been reported that more than 10 million people worldwide were affected by corneal damages. Although corneal transplantation is readily available in many countries, the supply of corneal tissue suitable for transplantation worldwide has never matched demand. In addition, the increasing number of laser in-situ keratomileusis (LASIK) operations, which disqualify donor tissue, has become a new threat to the availability of viable donor corneas. Even under the best conditions, donor grafts are typically variable in quality and usually fail due to immunological rejection or endothelial decompensation. Therefore, it is of great importance to develop alternative treatment regimens, including a tissue engineering approach, to replace conventional corneal transplantation [2-7].

Located at the anterior surface of the eye, the cornea is a highly specialized transparent avascular tissue that is composed of three functional layers: the outer stratified squamous epithelium, the inner endothelium, and the intermediate stromal layer. Among the total three layers, the corneal stroma comprises 90 percent of total thickness, with unique organization of the stromal collagen fibril, and is directly responsible for mechanical strength and transparency maintenance of the cornea. In addition, the stroma provides an anchoring structure to support epithelial and endothelial cells in their growth as well as performance of physiological functions.

Previous studies demonstrated the effectiveness of a tissue-engineering approach in restoring both the corneal epithelium [8-11] and endothelium [12-14] some of which have been applied clinically and have shown great promise in the recovery of corneal transparency. However, there have been few feasible reports on the reconstruction of tissue-engineered corneal stroma, which has been shown to be the most complicated and crucial part of constructing artificial corneas.

In a previous study, Hu et al. reported the construction of a nearly transparent corneal stromal layer in vivo using keratocytes isolated from corneal stroma [15]. However, considering that keratocytes are not readily accessible, of poor proliferation potential, and the harvesting of cells damages the donor cornea, we turned to finding an alternative source of seed cells for corneal stroma engineering. Adipose derived stem cells (ASCs) are a particularly useful source of mesenchymal stem cells (MSCs) due to their ease of harvest, robust proliferative capacity and multilineage potential [16]. The ability of ASCs to differentiate into adipocytes, chondrocytes and osteoblasts, for example, has been well documented [17]. In a recent study, Arnalich-Montiel et al. injected a cell suspension of human ASCs into a corneal stroma defect in a rabbit model. They found that human ASCs survived in the rabbit stroma for at least 12 weeks with restoration of the stromal structure [18], indicating that ASCs have the potential to differentiate into keratocytes in vivo. However, the utilization of a single-cell suspension without a scaffold for cell stabilization has drawbacks in cell leakage as well as uncontrollable cell distribution, especially in treating stromal defects along with epithelial injuries, which represent the most prevalent cases in the clinic compared with stromal injuries alone. Thus, it is of interest to study whether ASCs growing in a three-dimensional environment offered by scaffold could repair stromal defects both in structure and in function.

In addition to the study by Arnalich-Montiel et al., several other studies used MSCs derived from different kinds of human tissues, such as umbilical cord and corneal stroma, to restore injured or diseased corneal stroma in animal models [19,20].

Therefore, in our study, we grew autologous ASCs within a three-dimensional scaffold made of degradable PLGA in vitro to investigate the feasibility of repairing corneal stromal defects by transplantation of the cell-scaffold complex in a rabbit model.

## Materials and Methods

### Culture of rASCs in vitro and GFP labeling

New Zealand rabbits were purchased from Chenhang Experimental Rabbit Raising Farm (Shanghai, China). Forty-eight rabbits (3 months old) were included in this study. The experimental protocol was approved by the Animal Care and Experimental Committee of Shanghai Jiao Tong University School of Medicine in compliance with the tenets of ARVO Statement for the Use of Animals in Ophthalmic and Vision Research.

After the rabbits were anesthetized via an intramuscular injection of ketamine (15 mg/kg) and diazepam (4 mg/kg), a small piece of lipoaspirate was obtained from the nape region, cut into small fragments of about 1 mm^3^ and digested with 0.075% type I collagenase (Sigma Aldrich, St. Louis, MO) at 37°C for 30 minutes. Then the above mixture was centrifuged at 250 g for 5 min. The cells were collected and resuspended in DMEM-Low Glucose (DMEM-LG) culture medium, containing 10% FBS, 100U/mL streptomycin (Sigma-Aldrich, St. Louis, MO) and 100U/mL penicillin (Sigma-Aldrich, St. Louis, MO). The plates were maintained in a humidified atmosphere at 37 °C containing 95% air and 5% carbon dioxide. Following 48 h of incubation, non-adherent cells were removed by intensive washing with phosphate-buffered saline (PBS) and the medium was changed every 3 days. When reaching 80% confluence, cells were detached with trypsin-EDTA (0.25% trypsin and 0.2% EDTA) and subcultured. For cell labeling, cells at passage 3 were infected with recombinant retroviruses containing an enhanced green fluorescence protein (GFP) gene using a method previously published [21].

### Culture of cell-scaffold constructs

The polymer used in this study was polylactic-co-glycolic acid (PLGA) with aPLA/PGA ratio of 50:50 and the volumetric porosity of 90%, pore size of 300-500µm (Jinan Jianbokaiyuan Biomaterials Company, Shandong, China). The polymer was dissolved in chloroform, added to sieved sodium chloride particulate and stirred to obtain a paste-like polymer solution [22]. This ductile and deformable mixture was molded in a specially designed combined mold and evaporated in air for 24 h to remove the solvent. After vacuum drying at room temperature for at least 48 h, porogen leaching and drying, a PLGA scaffold with 7 mm in diameter and 220 µm in thickness was obtained. The porous scaffolds were kept desiccated until use. The disks were sterilized in ultraviolet light for 1 h and then soaked in DMEM containing 10% FBS overnight. GFP-labeled ASCs were collected and resuspended in culture medium at a density of 1×10^7^ cells/mL. About 20 µl of the cell suspension was seeded evenly onto each PLGA disk in 6-well plates. After incubation for 4 h with cell attachment, 4 mL of DMEM with 10% FBS was added into each well. The cell-PLGA constructs were subsequently cultured for 1 week in vitro before implantation and the culture media was replaced every 3 days.

### Proliferation and expansion of rASCs in the scaffold

At days 1, 2, 3, 4, 5, 6, 7 and 8 after seeding, the number of cells adhered to the discs was measured by a DNA assay, as previously reported [23]. Briefly, the cell-scaffold was washed with PBS twice, crushed and lysed with 0.5 mL proteinase K (0.5 mg/mL, Sigma Aldrich, St. Louis, MO) at 56°C overnight. After centrifugation at 12,000 g for 10 min, aliquots (40 µL) of the supernatants were extracted and mixed with 160 µL Hoechst 33258 dye solution (0.1 g/mL, Sigma Aldrich, St. Louis, MO) in black flat-bottomed 96-well plates (Corning Costar, USA). Absorbance of each sample was quantified using a Varioskan multi-mode detection reader (Thermo Electron Corp., USA) at a wavelength of 465 nm (the emission wavelength of 360 nm). Serial dilutions of a known concentration of rASCs were measured in the same way to draw a standard curve between absorbance and DNA content. The absorbance of non-seeded disc was also measured as a blank control, which was then subtracted from the corresponding samples.

In order to observe the adhesion and extracellular matrix (ECM) secretion, the cell-scaffold constructs that were incubated for 1 day and 7 days in vitro were collected for scanning electron microscopic examination. The PLGA scaffolds without cells were also observed as a blank control. Briefly, samples were fixed with 2.5% glutaraldehyde overnight, washed three times with PBS, and then dehydrated with an ethanol series and dried with hexamethyldisilazane. After sputter-coating with gold, the samples were viewed under the scanning electron microscope (Quanta 200 FEI, Netherlands).

A confocal laser scanning microscope (CLSM, Leica TCS SP2, Germany) was used for further visualization of the growth and distribution of rASCs on the constructs. For this purpose, the rASCs labeled with GFP were seeded on the scaffold as described previously, and the constructs were subjected to observation by a laser scanning confocal microscope (CLSM, Leica Microsystem, Germany) at 1 day and 7 days post-seeding.

### Stroma defect model in rabbits

Thirty rabbits were used to build stroma defect animal model using a previously described surgical procedure with modifications [15]. Contralateral unoperated corneas served as the control group. Briefly, under general anesthesia, a 100 mm deep, 6 mm diameter circular incision was made using a Barraquer trephine. A thin lamellar flap consisting of epithelial, Bowman layer and some anterior stroma was then constructed by an operating knife along a natural uniform stratum in the anterior corneal stroma. A stroma layer about 100µm below the flap was removed then. In experiment group, the graft of cell-scaffold construct was sutured into the recipient bed by using eight interrupted 10–0 nylon sutures; In control group, the flap was covered on to the stroma bed and fixed by cell-scaffold construct. After surgery, the rabbits were given topical 0.3% lincomycin ophthalmic drops TID for 1 month to prevent infection. Clinical examinations were followed up, including assess corneal optical clarity, neovascularization and degradation of grafts.

### Implantation of cell-scaffold constructs

After 1 week of incubation in vitro, the autologous rASCS-PLGA constructs were implanted into the corresponding rabbit corneal stroma (n=16). All procedures were performed on the right eyes of recipients. The recipient was anesthetized with an intravenous injection of sodium pentobarbital (30 mg/mL). The cell-scaffold construct was implanted by freehand pocket dissection to 40-60% of the depth of full-thickness cornea. The PLGA alone implantation group and the no graft group (n=16 each) were designed as control groups. The corneas of each group were grossly observed every week and photographed immediately after surgery and at 12 weeks and 24 weeks post-implantation.

### Histological and immunofluorescent analyses

For histological and immunofluorescent analysis, corneas from the experimental group and control groups were harvested at 12 (n=4 each) weeks and 24 (n=4 each) weeks post-implantation and dissected into halves. One half of each cornea was fixed in 4% paraformaldehyde for 24 h, embedded in paraffin, cut in 5 µm sections, and stained with hematoxylin and eosin (HE). The other section was snap-frozen in liquid nitrogen and embedded in optimal cutting temperature (OCT) compound (Tissue Tek, Elkhart, IN). 8-µm thick cross sections of corneal tissue were cut with a cryotome (model 3050; Leica, Deerfield, IL), mounted on glass slides and stored in -20°C for immunofluorescent staining.

After incubation with blocking buffer containing 5% bovine serum albumin (BSA, Invitrogen, USA) at 37°C for 30 min, the frozen cross-sections were incubated with goat anti-aldehyde-3-dehydrogenase1A1 (ALDH1A1) polyclonal antibody (1:100; Santa Cruz, Biotechnology Incorporation, Santa Cruz, CA), goat anti-keratocan polyclonal antibody (1:100, Santa Cruz Biotechnology Incorporation, Santa Cruz, CA) at 4°C overnight. Then they were washed 3 times with PBS, 10 min each time and incubated with rhodamin-conjugated secondary antibodies donkey anti-goat IgG antibody (1:300) at room temperature for 45 min. Finally, the sections were covered by cover slips with mounting fluid containing 5 g/mL of Hoechst 33258 (Sigma Aldrich, St. Louis, MO) and observed under the fluorescent microscope (Nikon Eclipse E600, Nikon, Japan).

### Collagen fiber measurement

The diameter and distribution of the collagen fibrils were measured at 12 weeks (n=4 each) and 24 weeks (n=4 each) post-implantation using a transmission electron microscope as previously described [15]. The samples were chosen in three different location of each corneal section and the diameter of 300 randomly selected collagen fibrils was measured in each sample. Statistical analyses were performed using ANOVA tests and statistical significance was set at *P* < 0.05.

### Traces of rASCs labeled with green fluorescence protein (GFP)

To confirm the survival and transformation of the rASCs in the corneal micro-environment, GFP expression in the corneal stroma was examined. After implantation for 6 months, the cornea was harvested and embedded in Tissue-Tek OCT compound (Sakura Finetek USA, Torrance, CA) for frozen sections, and GFP expression was detected under a fluorescent microscope (Nikon Eclipse E600, Nikon, Japan) for excitation/emission at 546/590 nm.

#### RNA isolation and real-time RT-PCR

Keratocan and ALDH1A1 are important markers of keratocyte. We used real-time RT-PCR method [24] to detect RNA of Keratocan and ALDH1A1 in different groups, in order to supply quantitative data to prove rASCs grew well on PLGA and expressed keratocyte markers in vivo. RNA was isolated using Trizol reagent (Invitrogen). RNA was precipitated with isopropanol and dissolved in diethylpyrocarbonatetreated distilled water. Total RNA (0.5 µg) was then reverse-transcribed using the random hexamer primer provided in first-strand cDNA synthesis kit. PCR primers for Keratocan, ALDH1A1 and GAPDH were as follows: for Keratocan, sense 5’-GAAGGCAAGGTGGTATAATGG-3’ and antisense 5’-ACAATCAAAGTCATCCGCAC-3’; for ALDH1A1 sense 5’- CAAAGACCTCGATAAAGCCGT-3’ and antisense 5’-ACCATATTCTCCCAGTTCTCGT-3’ for GAPDH, sense 5’-CGAGTACGTGGTGGAATC-3’ and antisense 5’- AGGGATGATGTTCTGGGC-3’. The real-time RT-PCR, which was performed in a final volume of 20 µl, consisted of 10 ng of reverse-transcribed total RNA, 167 nM forward and reverse primers, and 2×PCR Master Mix. The PCR was carried out in 96-well plates using an ABI Prism 7900HT Sequence Detection System. All tests were conducted in triplicate.

## Results

### Culture and labeling of rASCs in vitro

**Figure 1:**
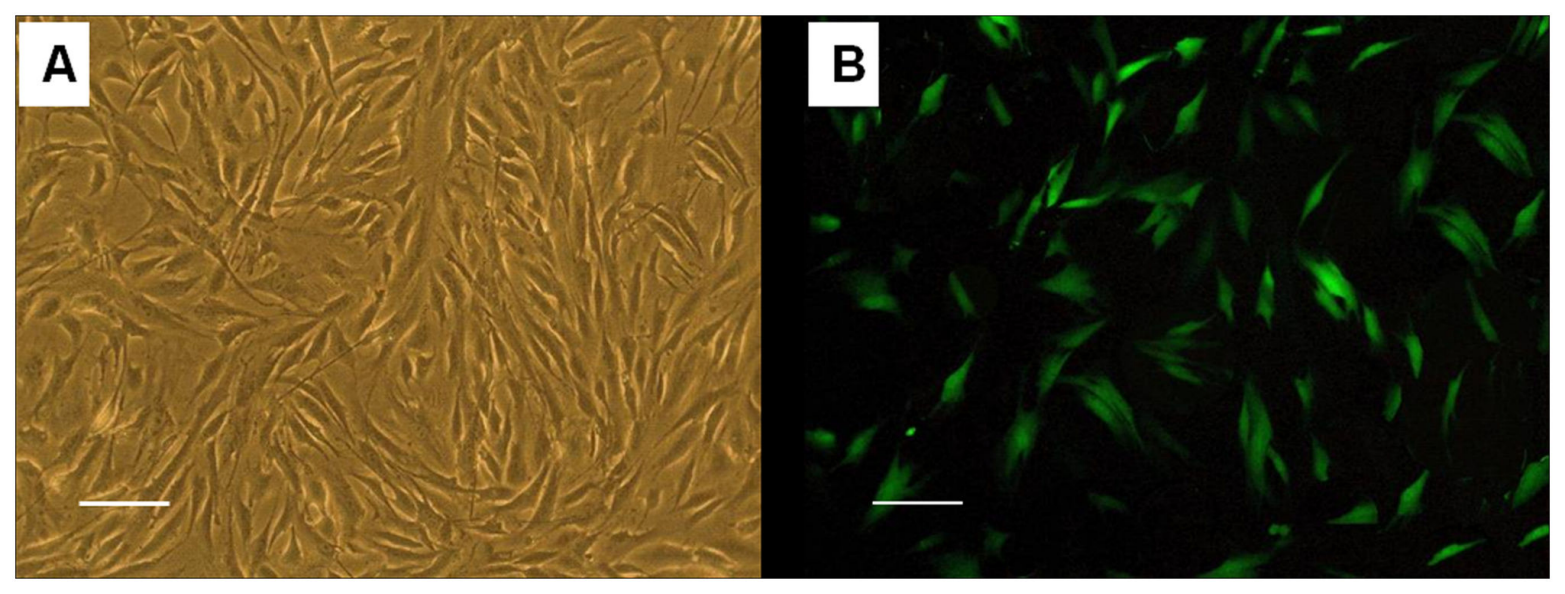
rASCs culture and GFP labeling. (A) rASCs at four passages, which appears to be a fibroblast-like uniform shape. (B) Fluorescence microscopic photograph of GFP labeled rASCs in Figure 1A. Most cells were GFP positive (Bar scales: 100 µm)..

**Figure 2:**
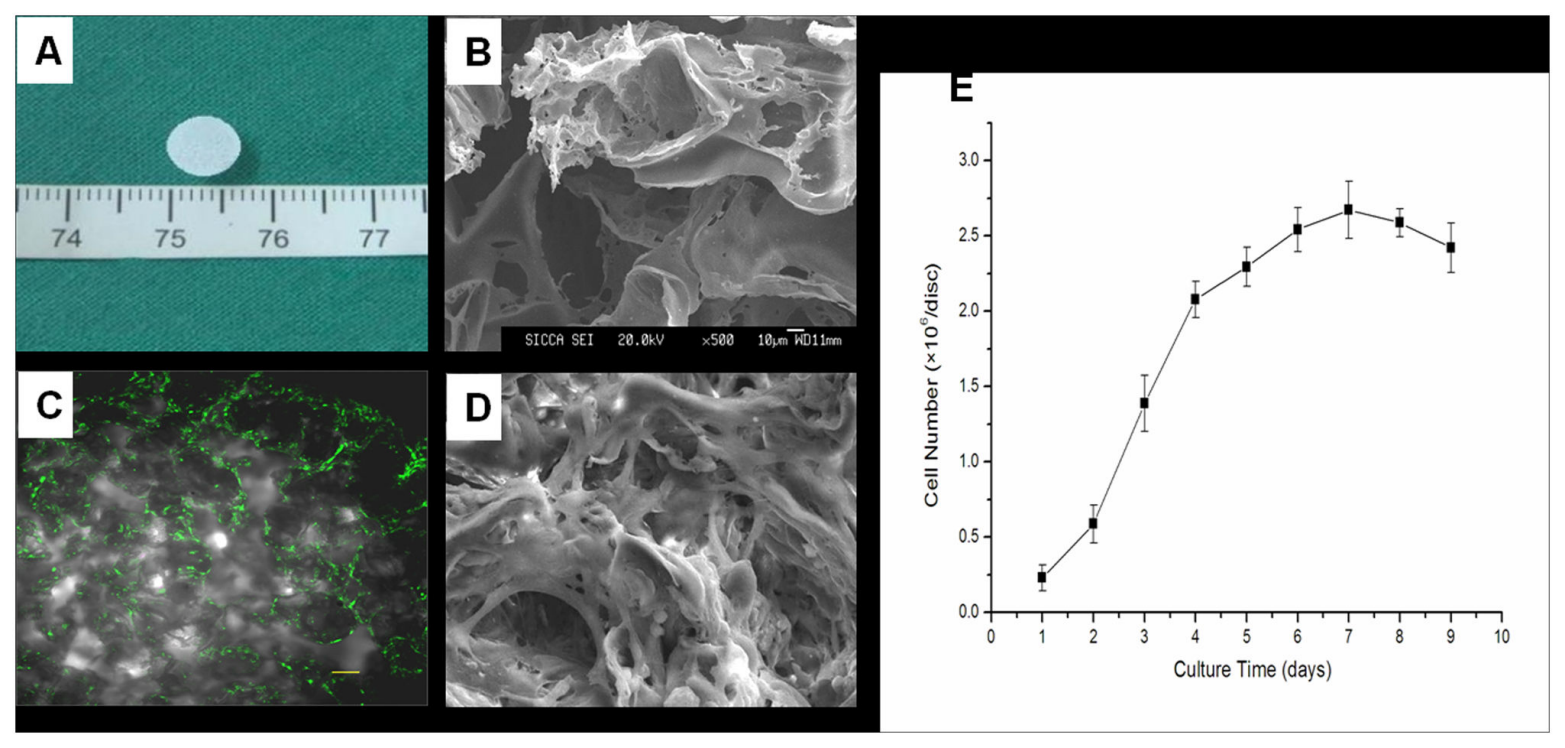
Proliferation and distribution of rASCs on a PLGA scaffold and secretion of the extracellular matrix (ECM). (A) Gross view of the PLGA scaffold with a 7 mm diameter. (B) Scanning electron micrograph (SEM) images of PLGA scaffold without cells. (C) The view of the rASCs and PLGA scaffold at 7 days was observed by confocal microscopy to reveal the cell distribution on the scaffold (Bar scales: 20 µm). (D) Attachment of rASCs on the same PLGA scaffold and deposition of the ECM are shown by SEM (magnification×500). (E) The quantitative evaluation of the proliferation of rASCs seeded on the PLGA scaffold determined by DNA assay using Hoechst33258 dye. Abbreviations: rASCs, rabbit adipose stem cells; PLGA, polylactic-co-glycolie acid; SEM, scanning electronic microscope.

rASCs subcultured to passage 3 in vitro acquired a fine fibroblast-like morphology (Figure 1A). After transfection with GFP adenovirus, about 50% of rASCs were observed to have GFP expression (Figure 1B). Cells were then harvested and seeded onto the PLGA scaffold for further in vitro cultivation for 7 days (Figure 2A). Confocal microscopy showed that rASCS were distributed evenly along the pores of the scaffold (Figure 2C). SEM microscopy showed that ASCs attached well on the scaffold and abundant ECM was deposited (Figure 2D). From the curve of cellular proliferation generated from the Hoechst33258 DNA assay, it was found that the cell number within the scaffold kept growing and reached a peak at 7 days (Figure 2E). These results indicated that the three-dimensional environment offered by the PLGA scaffold was suitable to support the attachment, proliferation and cellular matrix synthesis of the rASCs seeded within it.

### Clinical observation after surgery

**Figure 3:**
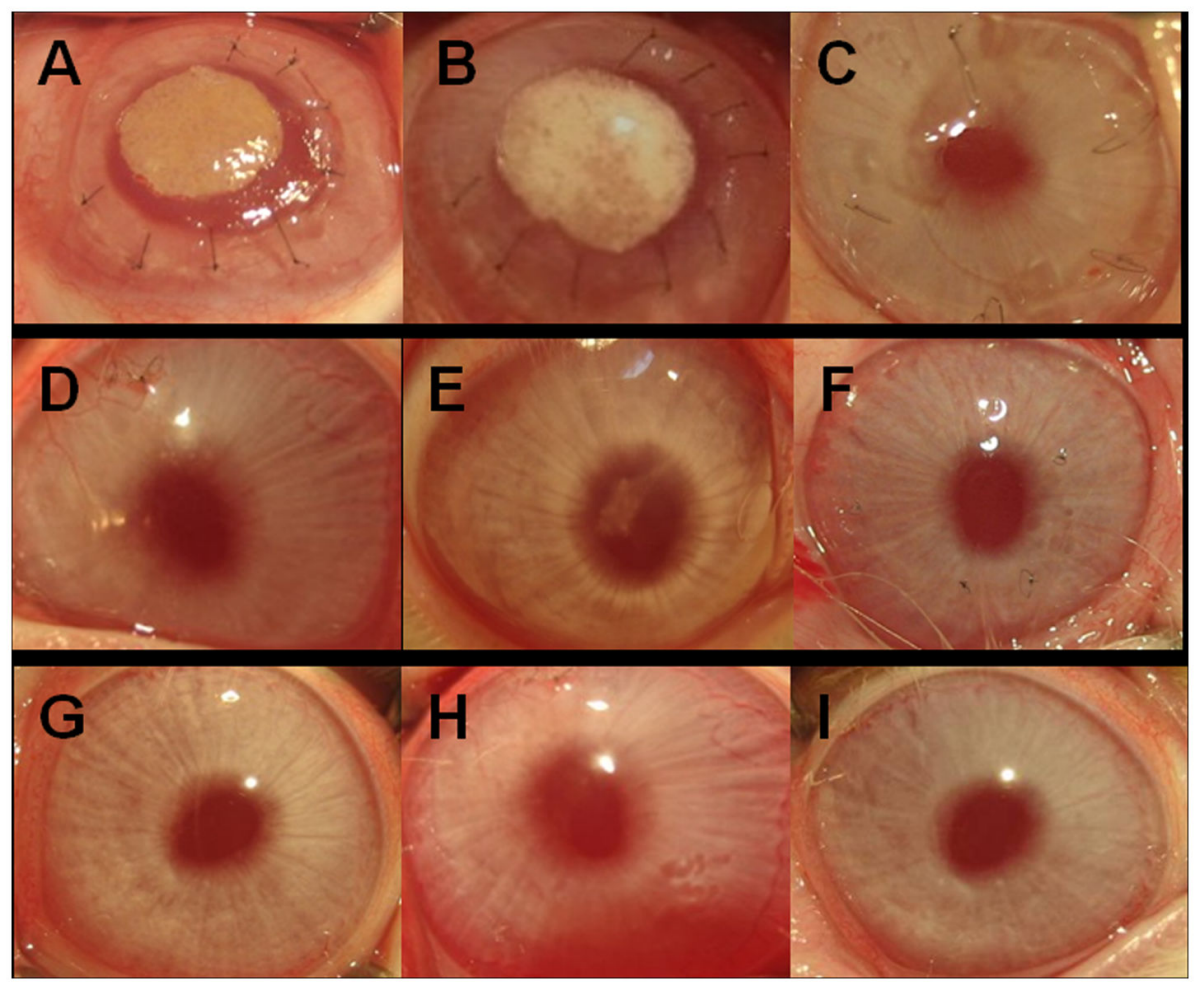
Gross view of the grafts in each group at 12 weeks and 24 weeks post-surgery. A, B, C: 0 weeks; D, E, F: 12 weeks; G, H, I: 24 weeks. A, D, G: Autologous rASCs+PLGA; B, E, H: PLGA alone; C, F, I: Only defect. At 12 weeks, the scaffold was mostly degraded and the corneas became nearly transparent in the group with implantation of the rASCs-PLGA construct while a partial scaffold still resides in the group with PLGA alone implantation. These two groups became transparent and showed no significant difference compared with the defect group and the normal cornea at 24 weeks. No inflammation or vascularization was found in all groups at 24 weeks after implantation.

After 1 week of incubation in vitro, the cell-PLGA grafts were implanted into the ablation stromal pocket. In the first few days after surgery, the graft looked similar to an opaque disc located in the center of the cornea. At 12 weeks post-transplantation, the scaffold was mostly degraded and the cornea became nearly transparent in the autologous rASCs-PLGA construct treated group (Figure 3D), which displayed a similar transparency as that in the non-treated group (Figure 3F). The remnant undegraded PLGA could still be discerned in the PLGA alone treated groups (Figure 3E). At 24 weeks after surgery, the corneas in the auto-rASCs (Figure 3G), PLGA alone (Figure 3H) and non-treated groups (Figure 3I) became almost as transparent as the normal cornea. In our study, the only defect animal model was built using scalpel, and the incision was tidy. So it was no wonder that the wound recovered well, no obvious scar presented. It is much different in clinical cases with cornea injuries or infection. All animals survived without side effects including corneal ulcer, corneal infection or flap displacement during the entire observation period.

### Histological evaluation of transplanted corneas

To confirm the outcome of gross observation, corneas from each group were collected at 12 weeks and 24 weeks after surgery and subjected to histological analysis.

**Figure 4:**
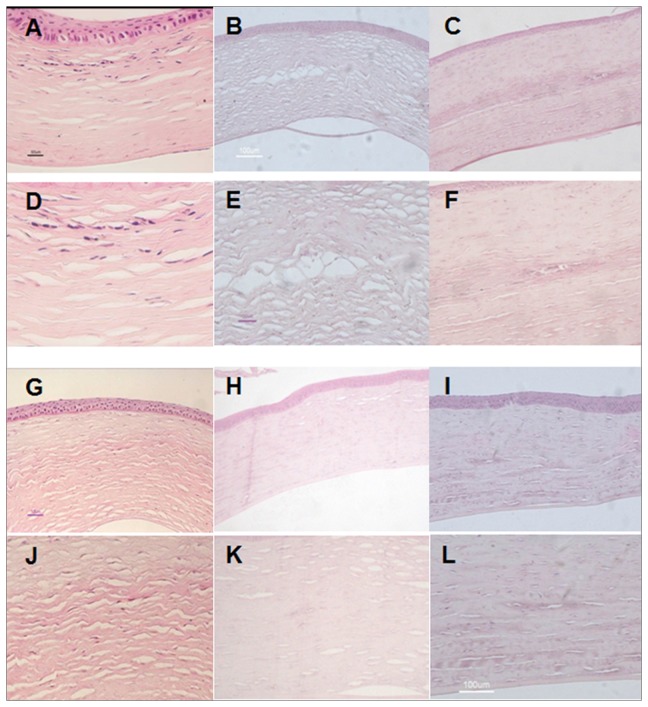
Histological analysis of tissue engineered grafts in each group at different time points. A, D, G, J: autologous rASCs+PLGA group; B, E, H, K: PLGA group; C, F, I, L: Only defect group; A-F: 12 week; G-L: 24 week. HE stained histological sections showed that the corneal epithelial cells and endothelial cells were intact in all groups. Corneas in the group with implantation of autologous rASCs-PLGA had been repaired by newly formed tissue, forming more native structures at 24 weeks than at 12 weeks post transplantation. In PLGA alone and only defect group, collagen remodeling was not successful. Corneas were thinner, collagen arrangement was not in good order, and scars existed (A-C, G-I: Magnification ×100; D-F, J-L: Magnification ×200)..

At 12 weeks after surgery, defects in corneas implanted with rASCs-PLGA constructs had been partially repaired with newly generated tissue, which could be clearly distinguished from surrounding normal tissue upon the existence of densely distributed cells (Figure 4A, D). By 24 weeks, the cornea re-acquired its native structure, which could be barely distinguished from the adjacent tissue (Figure 4G, J). The cells were observed to be scattered in the interface between collagen lamella and displayed a similar morphology to that of adjacent stromal keratocytes. Furthermore, In PLGA alone (Figure 4B, E, H, K) and only defect group (Figure 4C, F, I, L), collagen remodeling was not successful. Corneas were thinner, collagen arrangement was not in good order, and scars existed.

### Ultrastructure of stromal fibrils

**Figure 5:**
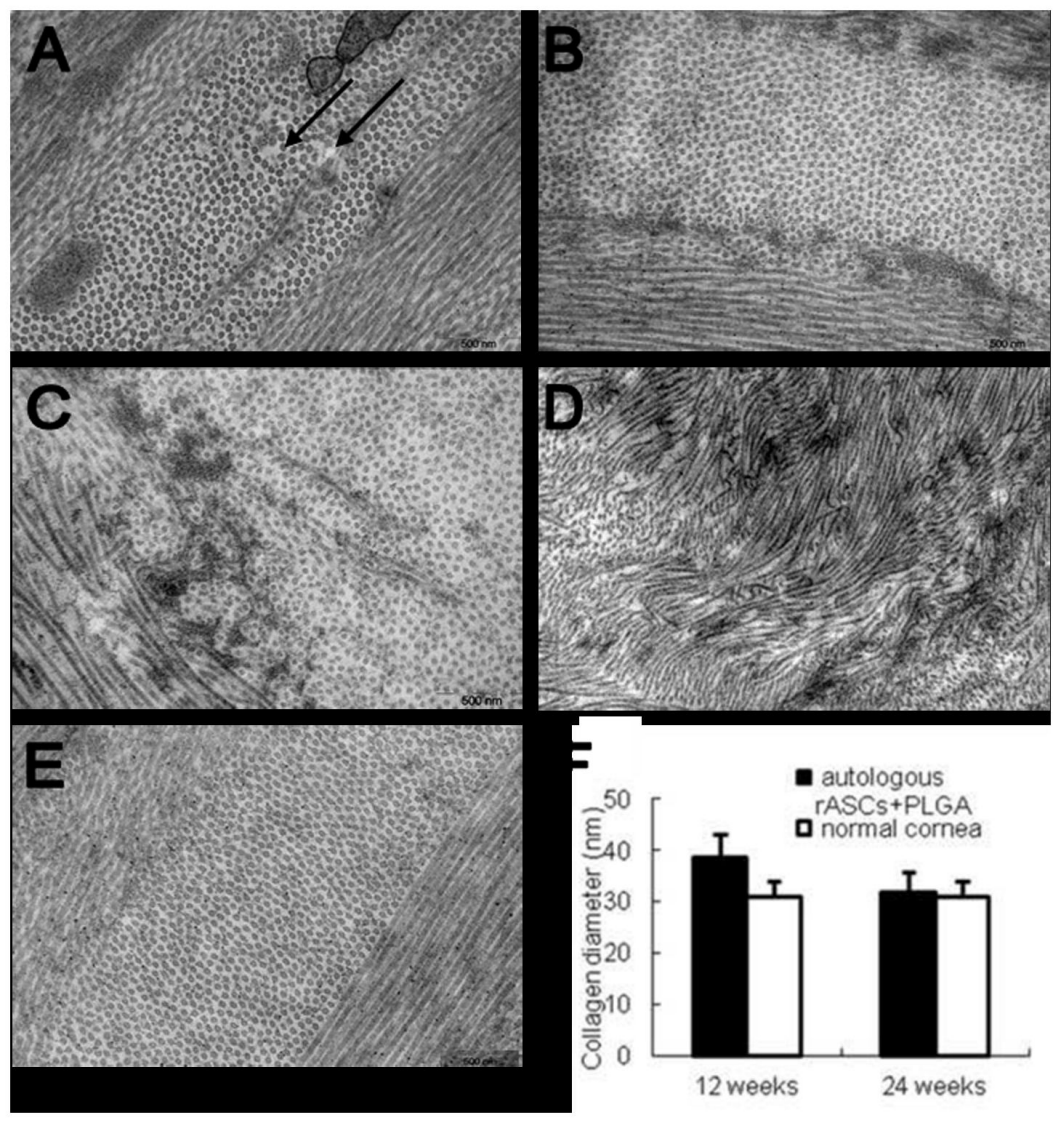
Distribution and diameter of collagen fibrils in different groups observed by transmission electron micrography. A, B: the rASCs+PLGA group autologous; C: PLGA group; D: Only defect group; A: 12 weeks post-implantation; B-D: 24 weeks post-implantation. (E) Distribution and diameter of collagen fibrils in normal cornea. Electron microscopic examination showed larger interfibrillar interval existed in the neoformative stromal matrix (arrow in Figure 5A) at 12 weeks, while normal lamellae containing regularly spaced fibrils formed at 24 weeks. Fibril diameter analysis showed neoformative stroma regained the identical diameter distribution of collagen fibrils to those of normal rabbit corneas in auto-rASCs group at 24 weeks (F) (Magnification ×12000)..

At 12 weeks after surgery, electron microscopy examination revealed that collagen fibrils in rASC treated corneas were distributed in a lamellae pattern associated with larger interfibrillar spacing existed in some region (arrow in Figure 5A). After 24 weeks, the stromal architecture in rASC treated rabbits was nearly identical to that in normal one (Figure 5B). In PLGA (Figure 5C) and only defect group (Figure 5D), lamellae structure could also be identified in stroma adjacent to the implanted layer. However, the implanted layer lacked normal fibril arrangement, being not parallel to one another and having a interlinked, tangled appearance 24 weeks post implantation. Therefore, it’s impossible to conduct the fibril diameter analysis for PLGA and only defect group. Figure 5E showed the distribution and diameter of collagen fibrils in normal cornea. By fibril diameter analysis, it was revealed that the diameter of collagen fibrils in the rASC group was larger than that of the normal cornea at 12 weeks (*P* < 0.05, Figure 5F), which regained their profile as identical to normal one after 24 weeks, with no significant difference (*P* > 0.05, Figure 5F).

### Survival of implanted rASCs, keratocan and ALDH analysis with immunofluorescence and realtime PCR

**Figure 6:**
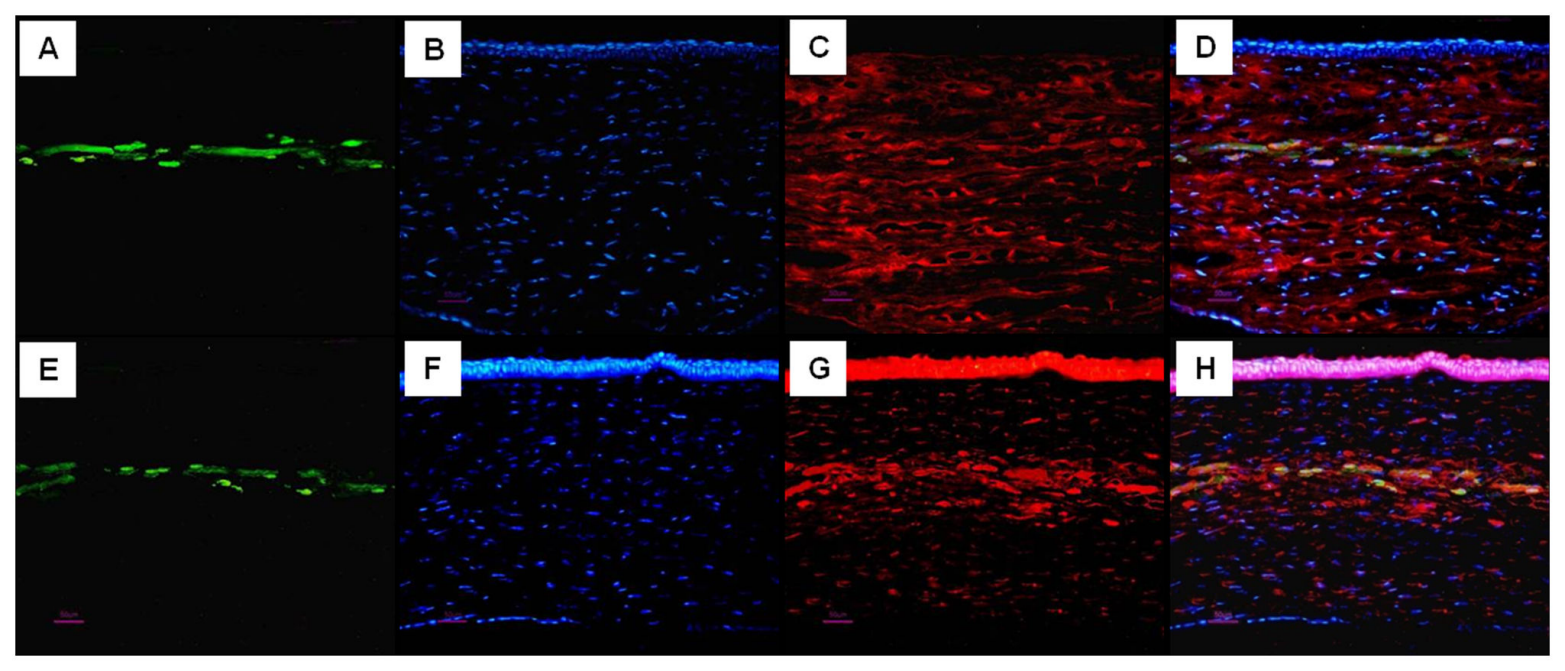
Survival of GFP-positive rASCs after 24 weeks of implantation and their differentiation to functional keratocytes. (A-H) corneas implanted with autologous rASCs-PLGA constructs; (A, E) GFP expression, (green color); (B, F) nuclei staining by hoechst33258 at the same section, (blue color); (C) expression of keratocyte specific proteoglycan keratocan at same section, (red color); (G) expression of ALDH1A1 at same section, (red color); (D, H) GFP expression, nuclei staining and keratocan, ALDH1A1 immunostaining are superimposed with Adobe Photoshop software, showing keratocyte proteoglycan production by implanted GFP-positive rASCs (Bar scales: 50 µm)..

To study whether implanted ASCs cells survived and secreted stromal specific extracellular matrix in vivo, GFP expression in the engineered corneas was examined using fluorescent microscopy. As shown in Figure 6, GFP-positive cells were detected in the corneas of the auto-rASC (Figure 6A, E) 24 weeks after implantation. No GFP-positive cells were observed in the corneas of the PLGA alone and non-treatment groups. Thus, this result indicated that autologous rASCs could survive for 24 weeks after implantation in vivo.

**Figure 7:**
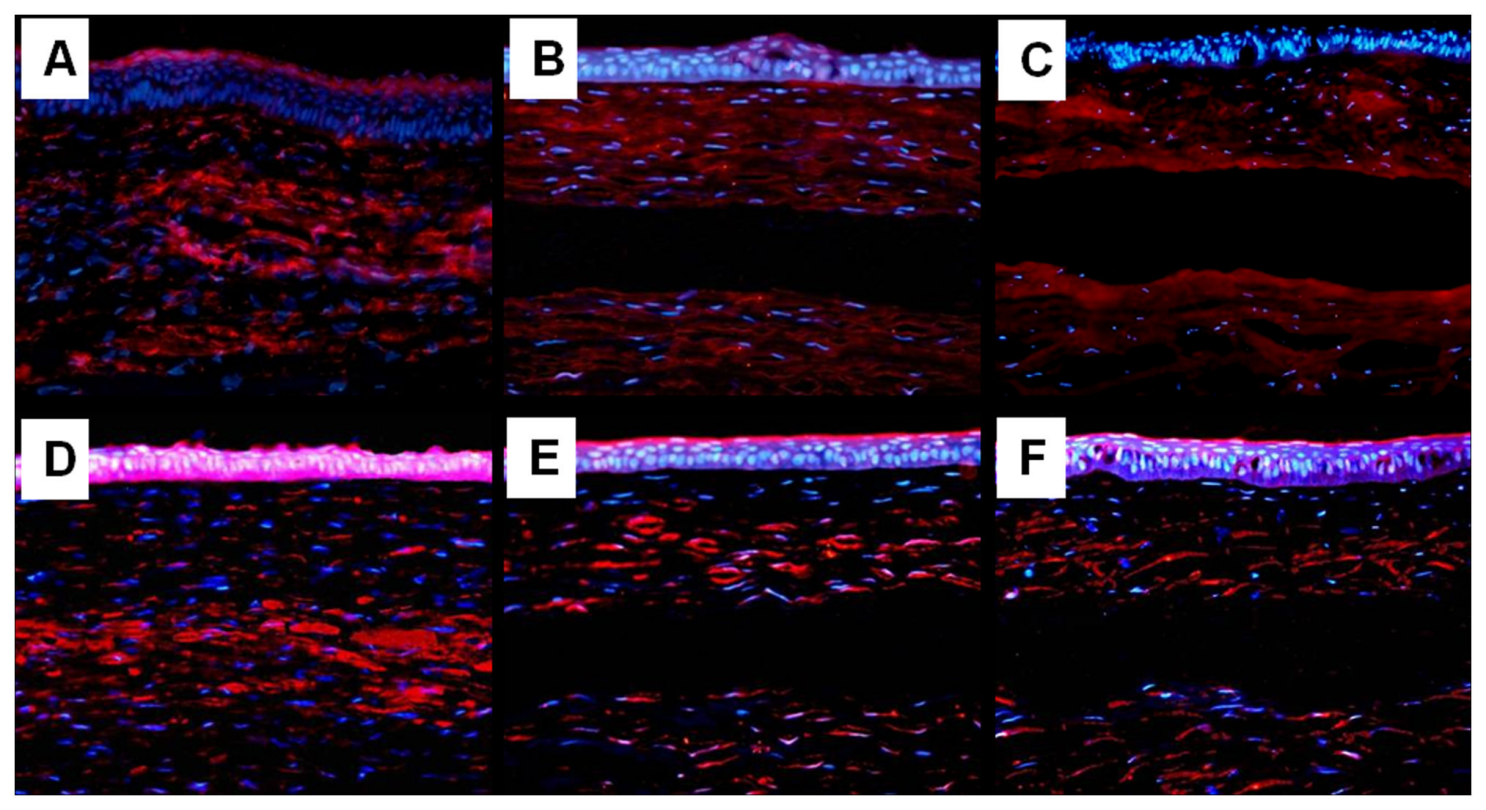
Rabbit keratocan and ALDH1A1 expression in each group at 24 weeks post-implantation. A, B, C: keratocan; D, E, F: ALDH1A1. (A, D) cornea implanted with autologous rASCs-PLGA constructs; (B, E) cornea implanted with PLGA alone; (C, F) cornea with defect only. (A-C) expression of keratocan; (D-F) expression of ALDH1A1. Both keratocan and ALDH1A1were positioned in the implanted layer of corneas implanted with the rASCs-PLGA complex, while the result was negative in corresponding layers in groups implanted with PLGA alone and defects only (Magnification×200)..

**Figure 8:**
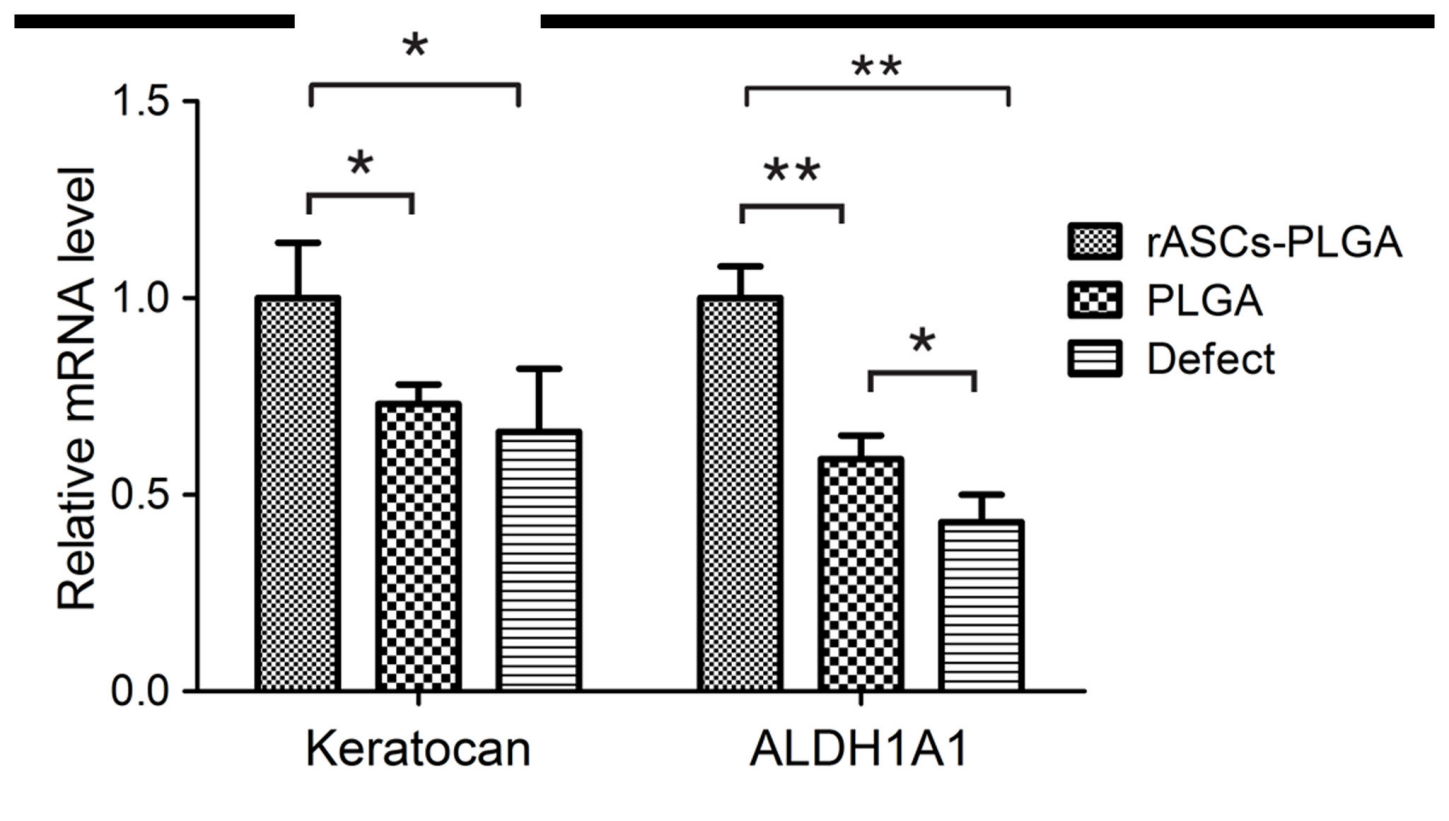
Messenger RNA levels of keratocan and ALDH1A1 were determined by real-time reverse transcriptase - polymerase chain reaction analysis. * p<0.05, **p<0.01.

To evaluate whether implanted rASCs assumed a keratocyte phenotype, expression of the keratocan, which has been accepted as a keratocyte specific marker, was detected by immunofluorescent staining. As shown in Figure 6, keratocan positive staining cells, which co-expressed GFP, could be observed in generated tissue in auto-rASC group. Moreover, co-localization of GFP and ALDH, functional stromal components, was also detected in auto-rASC treated corneas, respectively. On the contrary, keratocan and ALDH was absent in the corresponding position in the PLGA alone group (Figure 7B, E) and defect only group (Figure 7C, F). We used real-time RT-PCR method to detect RNA of Keratocan and ALDH1A1 in different groups. Figure 8 shows that keratocan and ALDH1A1 mRNA were highly expressed in rASCs-PLGA group compared to other two groups. The results showed that rASCs-PLGA could effectively repair keratocyte loss. Taken together, these results demonstrated that rASCs in the engineered corneas remained viable for long periods and differentiated into functional keratocytes in vivo.

## Discussion

Tissue engineering approach may provide alternative treatments for corneal diseases as well as substitutes for corneal injuries, but shortage of a seeding cell source can be a major obstacle in engineering corneal stroma, which represents more than 90% of the total thickness of the cornea. Considering the limited source of corneal stromal cells and concurrent damages to donor cornea when harvesting cells, to obtain a non-cornea derived cell source is urgent and significant in engineering corneal stroma for therapeutic purposes. In our study, ASCs were harvested from subcutaneous fat tissue, expanded, seeded within a PLGA scaffold and then implanted into the cornea to repair stromal defects. After 24 weeks, the damaged cornea, treated with the engineered ASCs-PLGA complex, restored their normal structure and transparency.

There have been several studies reported the effects that MSCs repaired corneal wound [23,25,26]. The animal models they built were thermal cauterzation or alkali burn model. They found that the MSCs (Bone marrow–derived mesenchymal stem cells or umbilical cord blood mesenchymal stem cells) were able to promote corneal epitheial and endothelial repairment. The seed cell and animal model they used were different from that of our study. Several previous studies have attempted a cell suspension based method for repairing injured corneal stroma by injecting cell suspension directly in situ [27,28]. However, injection of the cell solution in the clinic has the risk of cell leaking, especially when stromal damage is accompanied by loss of the epithelium. In addition, distribution of the injected cells could be easily altered under the influence of gravity and body position. Thus, we developed a method of seeding cells into a degradable scaffold, which could overcome the drawbacks of cell suspension injection. As a commonly used biomaterial, PLGA has been successfully applied in the fabrication of engineered tissue, such as bladder, cartilage and blood vessels [22,29,30]. Gomes et al [31] reported that they reconstructed cornea with tissue-engineered cell sheets composed of human immature dental pulp stem cells (hIDPSC) in a limbal stem cell deficiency rabbit model. They construct the animal model with a chemical burn method and superficial keratectomy was used to remove the damaged tissue. They found that hIDPSC sheet could successfully reconstruct corneal epithelium. In Gomes’ study, hIDPSC was found expressing mesenchymal stem cell (MSC) and several human embryonic stem (ES) cell markers. There were several differences between Gomes’ and our study. We built a corneal stroma defect animal model, and our destination was to repair the stroma defect by constructing tissue engineering corneal stroma applying rASCs. Besides, compared to IDPSC, ASCs were easier to get.

In our study, PLGA showed good cell compatibility with rASCs allowing cell attachment, proliferation and ECM deposition in vitro. PCR also showed that keratocan and ALDH1A1 highly expressed in rASCs-PLGA group compared to other groups, which means the transplantation model we built is effective. At 12 weeks post-implantation, it was observed that PLGA had been almost totally degraded with very rare remnant left, which scattered among the newly deposited collagen fibrils. No obvious inflammatory reaction was detected in either the autologous ASCs-PLGA complex or PLGA alone treated tissues.

It is well known that maintenance of a transparent cornea depends greatly on the unique architecture of collagen fibers in the stromal matrix, which is characterized by uniform diameters of collagen fibrils, similar interfibril separations and oriented fibrillar arrays within lamellae. In addition, during the healing process of the trephine wound in corneas, it has been widely observed that remarkable collagen orientation changes occur during the healing process of trephine wounds by x-ray diffraction [32,33]. In the present study, a similar process of collagen remodeling was observed in engineered stroma treated defects. At 24 weeks post-implantation, engineered stroma restored its collagen architecture in fibrillar organization, diameter size distribution and interfibrillar separation, resembling the normal transparent cornea.

Corneal transparence is dominated not only by the specific arrangement of collagen fibers, but also by deposition of the corneal stroma-associated keratan-sulfate proteoglycans and corneal crystallins, both of which locate among the spaces between collagen fibers. Keratocan is the keratan-sulfate proteoglycan solely synthesized by keratocytes, and has been shown to be a master regulator for the organization of collagen fibrils with uniform size and spacing [34,35]. As a major crystallin protein in rabbit corneas, ALDH1A1 is believed to contribute to corneal transparency through their light-scattering [24] and absorption effects [36,37]. Several studies demonstrated that a decrease in corneal transparency following injury was associated with the loss of ALDH1A1 expression in the stroma [24].

Thus, to further explore the mechanism responsible for transparency restoration in engineered stroma treated corneas, the expression of keratocan and ALDH1A1 was detected. In this study, keratocan and ALDH1A1 were detected as highly expressed in the area implanted with the rASCs-PLGA construct. No detectable expression of the keratocan and ALDH1A1 could be observed in PLGA treated alone stroma. These results indicate that, in addition to the specific organization of collagen fibers, deposition of stroma associated keratocan and ALDH1A1 proteoglycans is the other major issue responsible for transparency restoration in rASCs-PLGA treated corneas.

Even though a certain number of keratocyte-like cells were observed distributing within the rASCs-PLGA treated stromas at 24 weeks after transplantation, this does not provide insight as to whether these cells were recruited from surrounding normal tissues or were differentiated from the implanted rASCs. In order to further clarify the source of cells in the restored corneal stroma and the long-term fate of the ASCs implanted in vivo, rASCs were traced with transfection of GFP and the expression levels of keratocan and ALDH1A1 were detected. Positive expression of green fluorescence revealed that rASCs still survived in vivo after 24 weeks post-implantation. More importantly, co-expression of ALDH1A1 and keratocan was found in GFP labeled rASCs respectively, which demonstrated that surviving rASCs differentiated to the keratocyte phenotype. These results are consistent with a recent study by Liu et al in which they injected umbilical MSCs into the corneal stroma of lum-mice and found that these implanted cells assumed a keratocyte-like phenotype after implantation [30]. It has been speculated that complicated local environmental cues in the cornea, including growth factors, extracellular matrix and cell-cell interactions, may modulate the differentiation of stem cells into the keratocyte phenotype. However, the pattern in which these factors elicited their induction effect for ASCs differentiating into a keratocyte lineage remains to be investigated further.

Based on our results, we considered that adipose-derived ASCs could be a cell source for stromal repopulation and restoration in diseased corneas or for tissue engineering of corneal equivalents.

Taken together, our study demonstrated that autologous ASCs from adult rabbit nape adipose tissue could achieve effects on repairing corneal stromal defects and constructing tissue engineered corneal stroma, which provides an alternative treatment for corneal transplantation. However, there are still several limitations in using autologous ASCs as seeding cells, such as the painful liposuction process, duration of cell expansion and hard manipulation for quality control. Therefore, further research is necessary before ASCs could be used therapeutically in human corneal transplantation.
